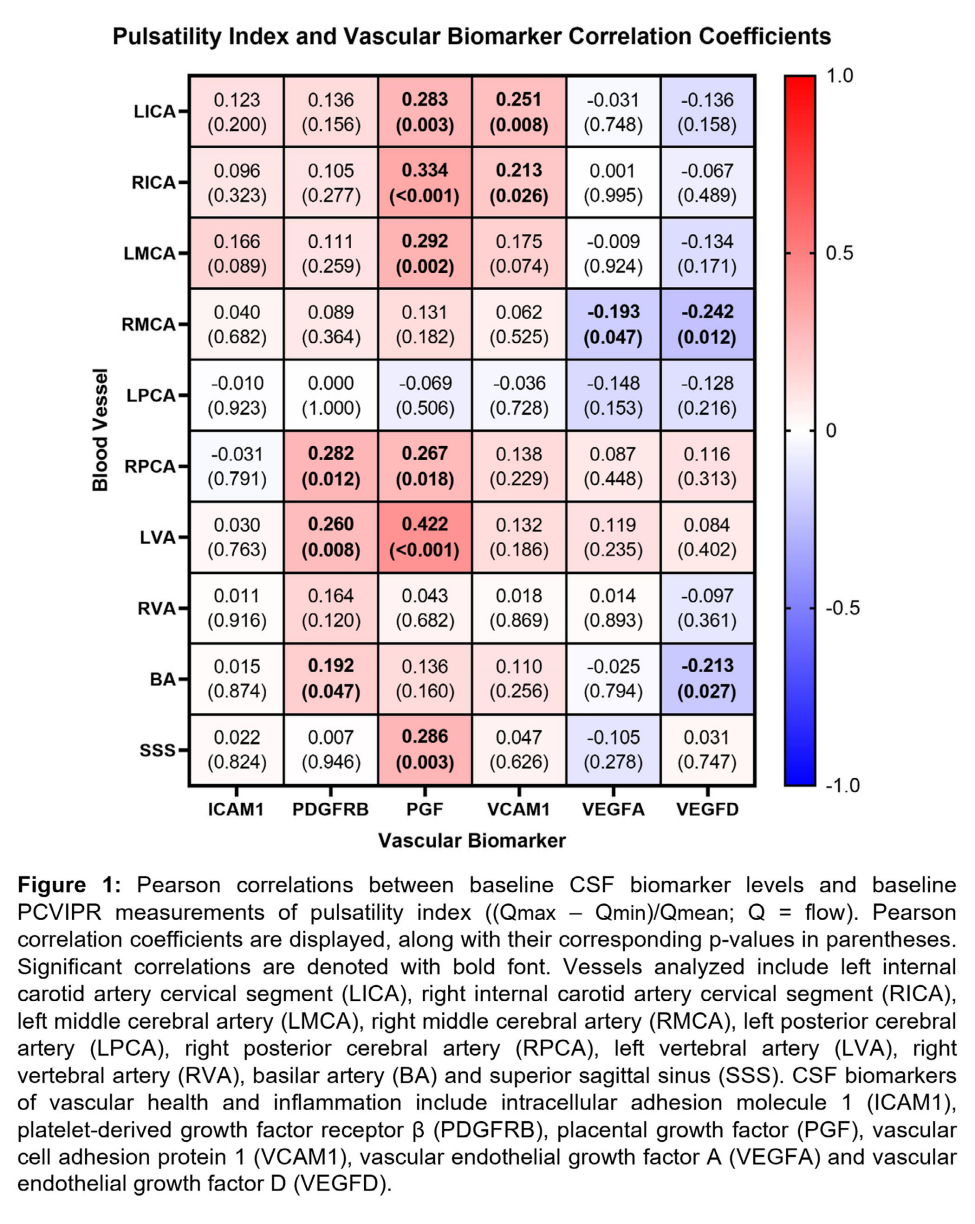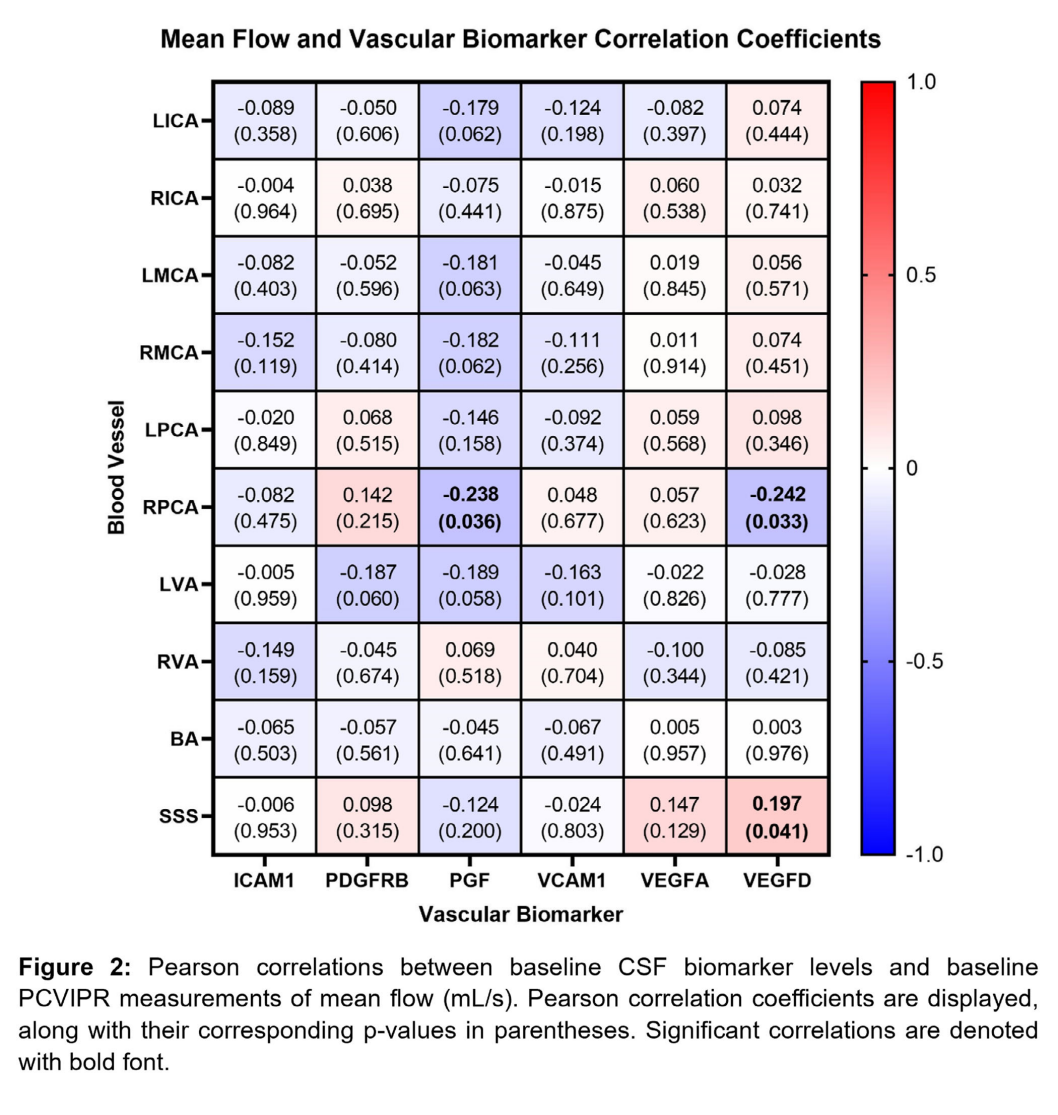# Effects of Icosapent Ethyl, *APOE* Genotype and CSF Biomarkers on 4D Flow MRI Metrics of Vascular Health in Cognitively Unimpaired Veterans

**DOI:** 10.1002/alz70856_101654

**Published:** 2025-12-25

**Authors:** Emma F Bublitz, Carol A. Van Hulle, Hannah Zylstra, Kate Cronin, Aleshia Cole, Kevin M. Johnson, Leonardo A. Rivera‐Rivera, Elena Beckman, Allison C Eierman, Rachael E. Wilson, Richard J. Chappell, Sanjay Asthana, Carey E. Gleason, Henrik Zetterberg, Sterling C Johnson, Cynthia M. Carlsson

**Affiliations:** ^1^ University of Wisconsin‐Madison, Madison, WI, USA; ^2^ William S. Middleton Memorial Veterans Hospital, Madison, WI, USA; ^3^ Wisconsin Alzheimer's Disease Research Center, University of Wisconsin School of Medicine and Public Health, Madison, WI, USA; ^4^ University of Wisconsin‐Madison Neuroscience Training Program, Madison, WI, USA; ^5^ University of Wisconsin School of Medicine and Public Health, Madison, WI, USA; ^6^ School of Medicine and Public Health, University of Wisconsin‐Madison, Madison, WI, USA; ^7^ Wisconsin Alzheimer's Disease Research Center, University of Wisconsin School of Medicine and Public Health, Madison, WI, USA; ^8^ Wisconsin Alzheimer's Disease Research Center, University of Wisconsin‐Madison, School of Medicine and Public Health, Madison, WI, USA; ^9^ Hong Kong Center for Neurodegenerative Diseases, Hong Kong, Science Park, China; ^10^ University College London, London, United Kingdom, London, United Kingdom; ^11^ Institute of Neuroscience and Physiology, University of Gothenburg, Gothenburg, Mölndal, Sweden

## Abstract

**Background:**

Veterans are more likely to develop vascular disease and Alzheimer's disease (AD) than non‐veterans. Icosapent ethyl (IPE), a form of the omega‐3 fatty acid eicosapentaenoic acid, reduces risk for major adverse cardiovascular events and lowers triglycerides. Improvement in vascular health may also reduce the risk of developing AD. 4D flow MRI, using PCVIPR (phase contrast vastly undersampled isotropic projection), offers direct study of blood flow and arterial stiffness in the brain. PCVIPR imaging may help uncover the effects of IPE on cerebrovascular health, and the relationship with *APOE* genotype and cerebrospinal fluid (CSF) biomarkers.

**Methods:**

The Brain Amyloid and Vascular Effects of Eicosapentaenoic Acid (BRAVE‐EPA) study enrolled VA‐eligible, cognitively unimpaired Veterans, ages 50‐75, at the Veteran's Hospital in Madison, Wisconsin. The study was a randomized, double‐blind, placebo‐controlled clinical trial of 4 g daily IPE (Vascepa®) treatment for 18 months. Participants had PCVIPR MRI, *APOE* genotyping and levels of CSF vascular health/inflammation biomarkers assayed with the NULISAseq CNS disease panel. PCVIPR imaging was completed on a 3T GE x750 scanner at baseline, month 9 and month 18. Image analysis was done with the Quantitative Velocity Tool in MATLAB. The PCVIPR measurements analyzed were mean blood flow (MF), pulsatility index (PI) and total cerebral blood flow (CBF; cervical internal carotid arteries + basilar artery). Interactions between variables were assessed with a Mann‐Whitney U test and using correlation analysis.

**Results:**

IPE treatment for 18 months did not significantly change MF, PI, or CBF measures (IPE: *n* = 41, placebo: *n* = 44; MF *p*‐value range: 0.064‐0.93; PI *p*‐value range: 0.061‐0.94; CBF *p*‐value: 0.33). *APOE* ε4 carriers (*n* = 28) and non‐carriers (*n* = 89) had similar baseline blood flow (MF *p*‐value range: 0.16‐0.92; PI *p*‐value range: 0.055‐0.98). In the full sample (*n* = 123), baseline CSF levels of vascular biomarkers were significantly correlated with baseline PI (*p*‐value range: <0.0001‐0.99; Figure 2). In contrast, few CSF vascular biomarkers were significantly correlated with MF (*p*‐value range: 0.032‐0.96; Figure 1).

**Conclusion:**

IPE treatment did not significantly impact PCVIPR measures of cerebral blood flow. Baseline vascular CSF biomarker levels, but not APOE ε4 carrier status, significantly correlated with baseline PCVIPR measures of PI and to a lesser extent MF.